# Electrically Tunable Tunneling and Spectral Response in WSe_2_/h‐BN/CdSe/Graphene Heterostructure

**DOI:** 10.1002/smll.73645

**Published:** 2026-05-06

**Authors:** Sang‐Hyeon Lee, Justice Agbeshie Teku, Min‐Hye Jeong, Jae‐Hyeon Ahn, Weon‐Sik Chae, Dohyun Kwak, Jong‐Soo Lee

**Affiliations:** ^1^ Department of Energy Science & Engineering Daegu Gyeongbuk Institute of Science and Technology (DGIST) Daegu Republic of Korea; ^2^ Division of Materials Science Korea Basic Science Institute Daejeon Republic of Korea; ^3^ Division of Nanotechnology Daegu Gyeongbuk Institute of Science and Technology (DGIST) Daegu Republic of Korea

**Keywords:** 2D materials, heterostructure, optoelectronic device, quantum dots, spectral responsivity, tunneling device

## Abstract

Mixed‐dimensional heterostructures consisting of zero‐ and two‐dimensional materials offer a promising platform for optoelectronic devices, as the versatility of material combination allows tunable optical properties. Bias‐induced approaches provide an additional means to tune the optical properties beyond the intrinsic band alignment of van der Waals junctions. Here, bias‐induced tunneling characteristics are achieved in vertically stacked WSe_2_/h‐BN/CdSe quantum dots/graphene heterostructures by employing the top graphene electrode to regulate carrier transport across the h‐BN barrier. The electrical analyses based on the Simmons approximation demonstrate tunneling‐mediated charge transfer through thin h‐BN layers and bias‐dependent modulation of the barrier height. Furthermore, tunneling‐induced exciton dissociation in WSe_2_ and CdSe QDs is observed through spectral responsivity and scanning photocurrent measurements. This work establishes a voltage‐dependent tunneling platform that enables deterministic control of carrier dynamics in mixed‐dimensional optoelectronic devices.

## Graphical abstract

In the WSe_2_/h‐BN/CdSe quantum dot/graphene heterostructure, the graphene voltage dynamically tunes the tunneling barrier and energy alignment across the h‐BN layer. The bias‐dependent Fermi‐level alignment enhances the tunneling density of states, leading to efficient extraction of photo‐excited electrons from QDs into WSe_2_ and graphene. Photocurrent mapping reveals spatially localized carrier separation governed by interfacial barrier modulation.

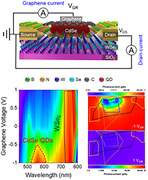

## 1 Introduction

Two‐dimensional (2D) transition metal dichalcogenides (TMDs) have garnered significant attention as building blocks for optoelectronic devices due to their atomically thin structure, tunable bandgaps, and intense light‐matter interactions [1, 2, 3]. In particular, van der Waals (vdW) heterostructures obtained by vertically stacking different 2D materials have provided a wide range of material combinations and design variety for optoelectronic applications [4, 5, 6]. However, the electronic band structures and their relative alignments in 2D–2D vdW heterostructures are inherently determined by the chosen material pairs [7, 8]. A mixed‐dimensional heterostructure consisting of zero‐dimensional (0D) semiconductor nanocrystals, commonly known as quantum dots (QDs), and 2D TMDs has emerged as an effective strategy to tailor optical and electronic properties [8, 9, 10, 11]. QDs exhibit unique optical features, including strong broadband absorption, size‐tunable bandgap, and high photoluminescence quantum yield [12, 13]. In 0D–2D heterostructures, QDs act as efficient photon absorbers and charge donors [14, 15, 16], thereby sensitizing TMDs to enhance their optical absorption, photoexcited carrier generation, and collection efficiency, ultimately improving the photoresponse and spectral selectivity of hybrid optoelectronic devices [17, 18].

The interface engineering in 0D–2D heterostructures plays a critical role in the interfacial charge transfer. Previous studies of 0D–2D heterostructures primarily relied on direct physical contact between 0D QDs and 2D semiconductors, which inherently limits the tunability of interfacial charge transfer [19, 20]. In van der Waals heterostructures, charge transfer at the interface is primarily governed by the intrinsic band alignment and atomic‐scale van der Waals gap [21], which constrains interfacial coupling and limits independent control over carrier injection, ultimately reducing charge transfer efficiency due to Fermi‐level pinning and trap‐assisted recombination [22, 23]. As an alternative strategy, an external electric field can be applied to van der Waals heterostructures to tune the interfacial junction. The electrical modulation enables a tunable transport‐mediated optical response, including carrier transport, spectral response, and photoresponse gain [24, 25, 26]. Recent advances have employed atomically thin insulating spacers, such as hexagonal boron nitride (h‐BN), which provide a new degree of freedom for engineering and tailoring the interfacial tunneling barrier [27]. In the 0D/insulator/2D configurations, the tunneling probability strongly relies on the tunneling barrier height, enabling selective modulation of carrier injection, suppression of defect‐mediated recombination, and precise control of the interfacial electrostatic environment.

In this work, we explore the carrier injection behavior from CdSe QDs into the WSe_2_ layer through the h‐BN tunneling barrier in WSe_2_/h‐BN/CdSe QDs/graphene heterostructures. By applying a voltage to the top graphene electrode, the interfacial barrier height is systematically modulated. Electrical transport measurements reveal tunneling‐mediated electron injections into WSe_2_ conducted under the application of bias to the graphene electrode, manifested as effective n‐type doping and corresponding variations in the normalized transconductance (Gm). Furthermore, under illumination, modulation of tunnel barrier height in the heterostructures controls the spectral absorption response and localized carrier generation. Complementary optical analyses, including spectral responsivity measurements and scanning photocurrent mapping, allow the identification of tunneling‐induced exciton dissociation and visualization of spatially localized carrier generation in the heterostructures. Finally, we demonstrate that electrically tunable tunneling facilitates engineered band alignment and enhanced charge transfer, leading to improved photodetector figures of merit. This strategy offers promising opportunities for optimizing photodetection in mixed‐dimensional systems.

## 2 Results and Discussion

### 2.1 Structure and Fabrication of the WSe_2_/h‐BN/CdSe QDs/Graphene Heterostructure

**FIGURE 1:**
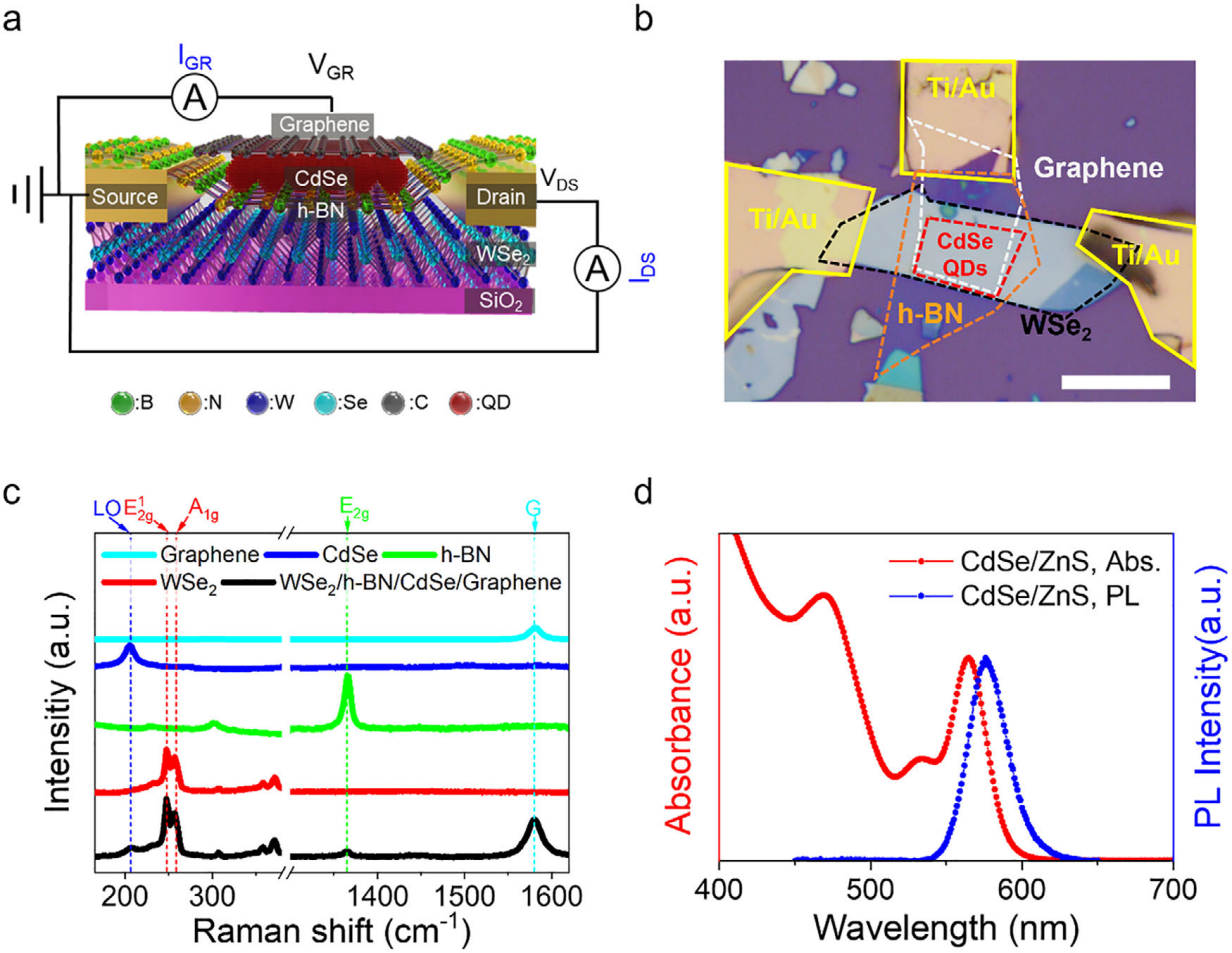
(a) Schematic illustration of the WSe_2_/h‐BN/CdSe QDs/graphene heterostructure fabricated on a SiO_2_/Si substrate. (b) Optical microscope image of the WSe_2_/h‐BN/CdSe QDs/graphene heterostructure. Scale bar: 10 µm. (c) Raman spectra of individual layers (graphene, CdSe QDs, h‐BN, WSe_2_) and the stacked heterostructure, measured with a 532 nm excitation laser. Characteristic phonon modes (E1_2 g_, A_1g_, LO, and G) show negligible shifts, indicating minimal strain and weak interlayer coupling. (d) Absorbance and PL spectra of CdSe/ZnS QDs showing the first excitonic absorption peak at 563 nm and emission at 577 nm (FWHM ≈ 30 nm).

Figure 1a illustrates a WSe_2_/h‐BN/CdSe QDs/graphene heterostructure designed to investigate tunneling‐mediated charge transfer across the h‐BN barrier. The WSe_2_ channel layer was transferred onto a SiO_2_/Si substrate via the dry transfer method [28, 29]. The source‐drain electrodes were then patterned, followed by the deposition of Ti/Au (10/40 nm). The thin h‐BN was vertically stacked as the tunneling barrier between the CdSe QDs and the WSe_2_ channel. The CdSe QDs, which act as the light absorber, were coated onto the active area pre‐patterned using e‐beam lithography. The patterned CdSe QD films were fabricated to avoid direct contact with the source/drain electrodes, suppressing undesired current flow through the QDs themselves. This study utilizes a CdSe/ZnS core–shell structure, in which the CdSe core is encapsulated with a thin ZnS shell to provide effective surface passivation while allowing efficient carrier transport into the adjacent channel. Lastly, we transferred graphene onto the top of the heterostructure. The application of voltage to the top graphene electrode (V_GR_) allows a tunable control of carrier injection from CdSe QDs into the WSe_2_ channel across the h‐BN tunnel barrier, governing the tunneling current and transport pathway at the QDs/WSe_2_ interface. Figure 1b presents an optical microscope image of the fabricated device. The WSe_2_ flake forms a well‐defined channel between the source and drain electrodes, while the CdSe QDs are confined to the central region of the channel. This optical image shows the precise spatial alignment of each component in the stacked heterostructure, which is essential for independent probing of the drain (I_DS_) and graphene currents (I_GR_) in the subsequent electrical and optoelectronic measurements. Figure 1c shows Raman spectra for pristine graphene, CdSe/ZnS QDs, h‐BN, WSe_2_, and the heterostructure, respectively. Raman spectroscopy provides information on strain, interlayer coupling, and charge transfer [30, 31, 32, 33]. Raman spectra of the heterostructure resolve the layer‐specific phonon modes, CdSe LO (∼210 cm^−^^1^, longitudinal optical vibration of Cd─Se bonds), h‐BN E_2g_ (∼1366 cm^−^^1^, in‐plane B─N stretching), E^1^_2g_, and A_1g_ of WSe_2_ (∼250/260 cm^−^^1^, in‐plane and out‐of‐plane lattice vibrations) at their reference positions [34, 35]. In particular, the characteristic modes of WSe_2_ (E1_2 g_ and A_1g_) appear at the same positions as those in the pristine sample, indicating the absence of any observable shift or broadening. This result indicates that the insertion of the h‐BN layer, as an atomically flat and chemically inert space, effectively suppresses interfacial strain and prevents phonon–electron coupling between WSe_2_ and the CdSe QDs. The preservation of the intrinsic lattice dynamics in the stacked configuration enables clear separation between structural effects and electric field‐induced band modulation in subsequent analyses. Figure 1d shows the photoluminescence (PL) and absorption spectra of the CdSe/ZnS QDs. The PL spectrum exhibits an emission peak centered at 577 nm and an excitonic absorption peak at 563 nm. The chemical composition of the CdSe QDs is investigated via X‐ray photoelectron spectroscopy (XPS). As shown in Figure S2a,b, the Cd 3d spectrum exhibits a dominant Cd 3d_5/2_ peak at 411.8 eV, consistent with Cd^2+^ in CdSe, while the Se 3d spectrum displays a Se 3d_5/2_ peak at 54.5 eV, corresponding to Se^2−^ in CdSe. Absence of additional characteristic features indicates minimal surface oxidation, confirming the formation of stoichiometric CdSe with well‐defined chemical states. Figure S3 shows cross‐sectional high‐resolution scanning transmission electron microscopy (STEM) images of the WSe_2_/h‐BN/CdSe QD heterostructure. The atomically resolved interface clearly distinguishes the layered WSe_2_ (18 nm) and ultrathin h‐BN (2.5 nm), forming a clean and well‐defined van der Waals junction. A uniform CdSe QD film composed of densely packed nanocrystals with an average size of approximately 3.8 nm is observed.

### 2.2 Structural and Electrical Control of the h‐BN Tunneling Barrier

**FIGURE 2:**
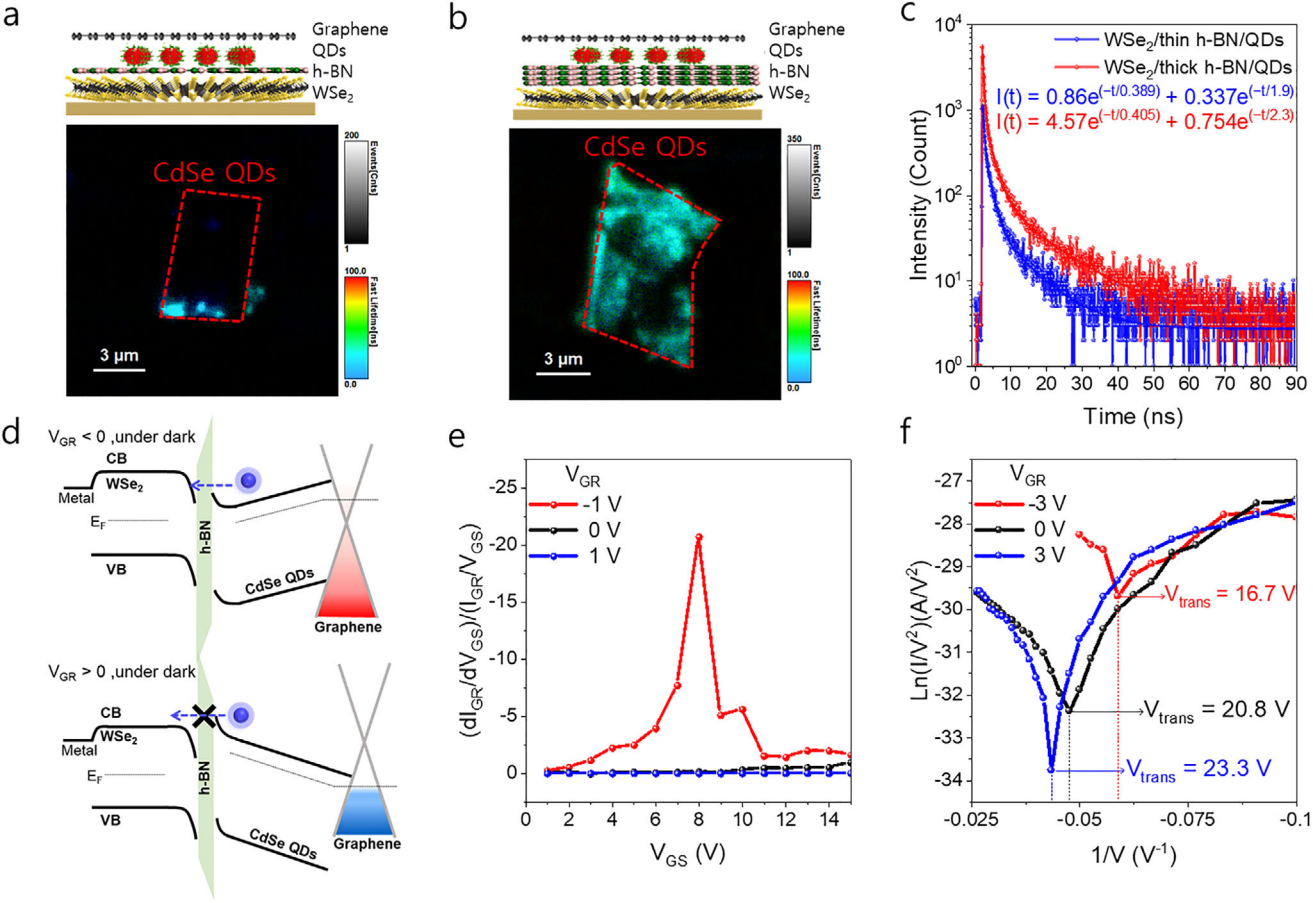
(a,b) Time‐resolved photoluminescence (TR‐PL) mapping images of CdSe QDs in heterostructures with thin (∼3 nm) and thick (∼30 nm) h‐BN barriers, measured under 470 nm pulsed excitation (repetition = 2 MHz). (c) TR‐PL decay profiles fitted with multi‐exponential functions, showing shorter lifetimes for the thin barrier, evidencing efficient tunneling‐mediated carrier extraction. (d) Schematic band diagrams illustrating barrier modulation under V_GR_ < 0 V and V_GR_ > 0 V under dark. (e) Normalized differential conductance (dI_GR_/dV_GS_)/(I_GR_/V_GS_) curves at V_GR_ = −1, 0, and +1 V showing a prominent conductance peak at V_GR_ = −1 V, corresponding to DOS‐assisted tunneling alignment. (f) Fowler–Nordheim plots measured at room temperature.

We investigated the charge coupling in the heterostructure with thin and thick h‐BN because the charge transfer across a h‐BN spacer relies on its thickness. The time‐resolved photoluminescence (TR‐PL) measurements were conducted as a function of h‐BN barrier thicknesses. TR‐PL provides quantitative information on the emission lifetime during radiative recombination of photoexcited carriers, enabling evaluation of the underlying charge–transfer dynamics [20]. Figure 2a,b shows TR‐PL mapping images of the heterostructures with a thin h‐BN barrier (∼3 nm) and a thick h‐BN barrier (∼30 nm), respectively. The TR‐PL mapping images represent spatial visualization of the carrier lifetimes and photon counts across the sample. The sample with a thinner h‐BN barrier exhibits reduced photon counts and faster carrier lifetimes, whereas the sample with a thicker barrier leads to increased photon emission and prolonged recombination dynamics. Figure 2c shows the corresponding decay profiles fitted using tri‐exponential and four‐exponential models, yielding lifetime components of 0.389, 1.9, and 9.9 ns for the thin‐barrier device, and 0.405, 2.3, 12, and 0.008 ns for the thick‐barrier device. The lifetimes are assigned to direct exciton recombination (τ_1_), recombination via shallow trap states (τ_2_), and recombination through deep trap states (τ_3_) [36]. The amplitude of the short‐lived component (A_1_) is significantly lower in the thin‐barrier structure (0.861 kCnts) compared to the thick‐barrier structure (4.57 kCnts) in Table 1. The result indicates efficient tunneling‐mediated charge transfer from CdSe/ZnS QDs into WSe_2_ across the thin h‐BN barrier, which suppresses radiative recombination within QDs. In contrast, tunneling in the thick‐barrier structure is strongly inhibited, resulting in a higher ratio of direct radiative recombination. Furthermore, the trap‐assisted recombination in which photogenerated carriers are readily captured at interfacial trap states plays a more dominant role in the thin‐barrier structure (A_2_ = 27.1% and A_3_ = 3.5%) than in the thick‐barrier structure (A_2_ = 14% and A_3_ = 1.8%).

**TABLE 1:** TRPL fitting parameters for WSe_2_/h‐BN/CdSe QD heterostructures.

Sample	A_1_ (kCnts)	Tau_1_ (ns)	A_2_ (kCnts)	Tau_2_ (ns)	A_3_ (kCnts)	Tau_3_ (ns)	A_4_ (kCnts)	Tau_4_ (ns)	<Tau> (ns)
Thin h‐BN	0.861	0.389	0.337	1.9	0.044	9.9	—	—	4
Thick h‐BN	4.57	0.405	0.754	2.3	0.0991	12	0.00801	98	17

Figure 2d schematically illustrates the band alignment of the WSe_2_/h‐BN/CdSe QDs/graphene heterostructure under dark conditions at a positive and a negative V_GR_. The interlayer interaction between WSe_2_ and CdSe forms a type‐II staggered band alignment [37]. In this heterostructure, an h‐BN layer inserted between the CdSe QDs and the WSe_2_ layer suppresses the static charge transfer due to its wide bandgap. Furthermore, the integration of a top graphene electrode allows for electrically tunable tunneling. When a negative V_GR_ is applied to the graphene electrode, its Fermi level shifts upward, inducing a tilted potential profile across the h‐BN barrier. This band bending reduces the effective tunneling barrier in the CdSe QDs, enhancing the probability of tunneling‐mediated electron injection toward the WSe_2_ channel. The majority carriers in CdSe QDs are electrons [38], while the contribution of holes is negligible. In contrast, an application of positive V_GR_ to the graphene electrode shifts its Fermi level downward, suppressing electron transfer from the QDs to WSe_2_ layer by increasing the tunneling barrier. Figure 2e presents the normalized differential conductance, defined as (dI_GR_/dV_GS_)/(I_GR_/V_GS_), of the WSe_2_/h‐BN/CdSe QDs/graphene heterostructure as a function of V_GS_ under different graphene voltage conditions. A pronounced conductance peak appears only at V_GR_ = −1 V, located near V_GS_ = 8 V, whereas the spectra for V_GR_ = 0 and 1 V remain nearly featureless. This behavior is attributed to the voltage‐induced lowering of the tunneling barrier, accompanied by Fermi‐level alignment among graphene, discrete QD states, and the WSe_2_ conduction band edge, collectively enhancing the density of available tunneling states. This band alignment gives rise to a pronounced increase in the interfacial DOS, which is manifested as a sharp conductance peak and promotes efficient electron transfer from the CdSe QDs into the WSe_2_ channel across the h‐BN barrier, consistent with DOS‐mediated tunneling models reported in previous tunneling spectroscopy studies [39, 40, 41]. Figure 2f presents the Fowler–Nordheim (FN) plots at V_GR_ = −3, 0, and 3 V, respectively. The observed behavior indicates a transition from direct tunneling (DT) to Fowler–Nordheim tunneling (FNT), implying voltage‐dependent modulation of the tunneling barrier [39, 40, 42]. The inflection point in each curve reflects the transition voltage (V_trans_) between FN tunneling and direct tunneling, which is defined as V_trans_ = ϕ (barrier height)/e (elementary charge) [43]. The extracted V_trans_ values are 16.7, 20.8, and 23.3 V at V_GR_ = −3, 0, and 3 V, respectively. This result indicates that the tunneling barrier height is significantly reduced at V_GR_ = −3 V compared with the other voltage conditions, consistent with the band alignment induced by negative voltage. In the thick h‐BN device (∼35 nm), the *I–V* curves exhibit a systematic bias‐dependent shift rather than tunneling‐related modulation, indicating that the transport is dominated by leakage current rather than quantum tunneling, as shown in Figure S9.

### 2.3 Tunneling‐Induced Exciton Dissociation and Charge Transfer

**FIGURE 3:**
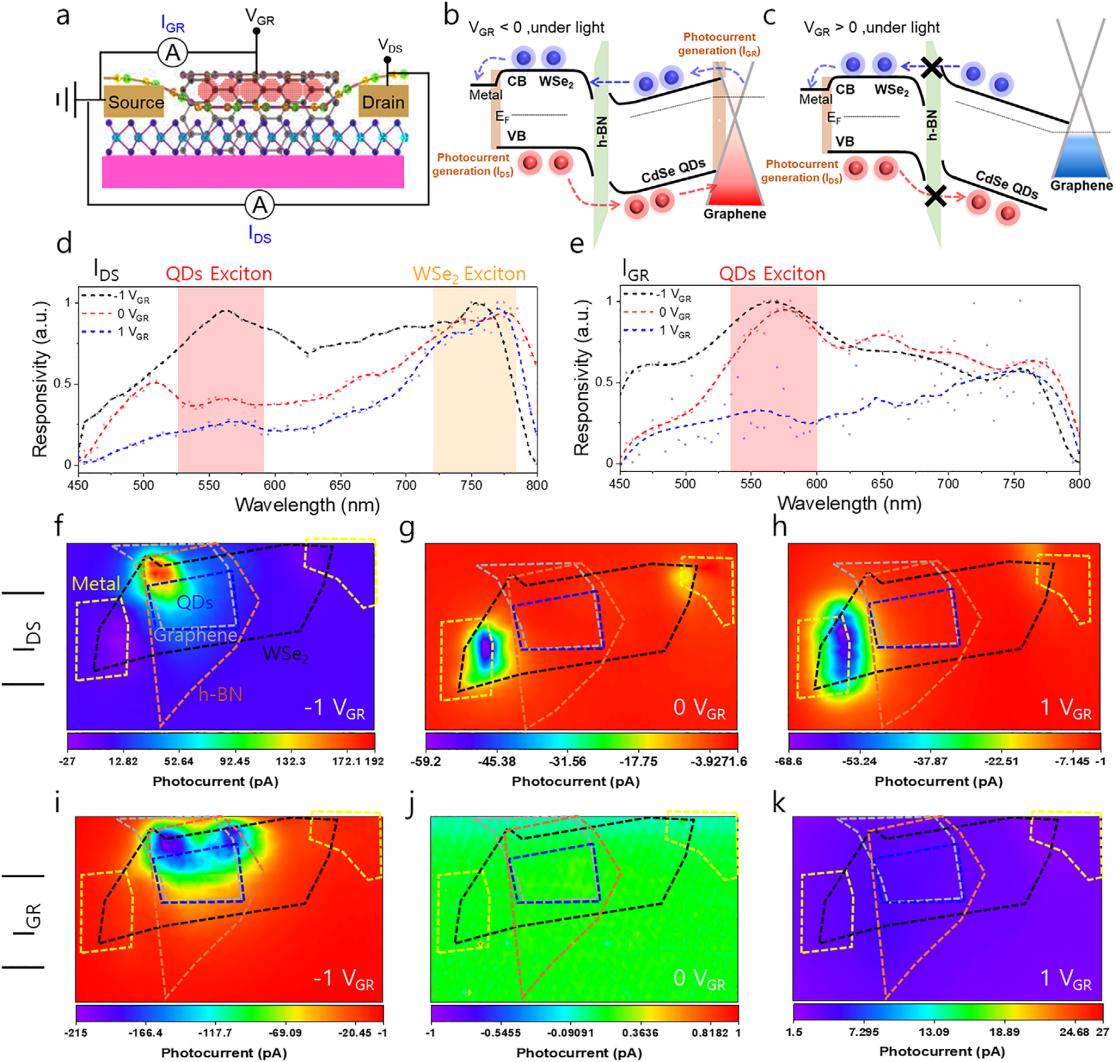
(a) Schematic measurement configuration for independent drain and graphene current detection. (b,c) Energy band diagrams illustrating charge–transfer pathways at V_GR_ < 0 V (b) and V_GR_ > 0 V (c) under light. (d,e) Spectral responsivity of drain and graphene current measured under monochromatic illumination (450–800 nm, step = 10 nm). Distinct peaks at ∼550 nm (CdSe QD exciton) and ∼760 nm (WSe_2_ A−exciton) appear under negative V_GR_. (f–h) Drain‐photocurrent maps obtained at V_GR_ = −1 (f), 0 (g), and 1 V. (h) using a 532 nm laser (0.37 W/ cm^2^). (i–k) Graphene‐photocurrent maps under identical conditions showing strong photo‐carrier separation at −1 V and suppressed response at 0 and 1 V.

The CdSe QDs exhibit strong optical absorption and efficient photocarrier generation under illumination. When incorporated into a tunneling heterostructure, these photocarriers participate in interfacial charge transfer processes that significantly modulate the charge transport characteristics. We investigated the photoresponse of the WSe_2_/h‐BN/CdSe QDs/graphene heterostructure under various V_GR_ conditions and illumination wavelengths. The interfacial tunneling pathway efficiently mediates carrier transfer between the 2 and 0D layers, leading to pronounced photocurrent modulation as a function of V_GR_. The QD‐derived carriers actively govern the overall photoresponse of the tunneling device. Figure 3a schematically illustrates the measurement configuration in which photocurrents at the drain (I_DS_) and graphene electrode (I_GR_) are independently recorded. Here, I_GR_ denotes the current measured at the graphene electrode, including both dark current and photocurrent generated by photo‐excited carriers extracted into graphene. By separating the I_DS_ from the I_GR_, carriers injected into the WSe_2_ channel can be distinguished from those directly extracted by the graphene electrode. This electrode‐resolved current measurement provides a basis for analyzing QD‐derived charge‐transfer pathways across the h‐BN barrier.

Figure 3b,c depicts the schematic band diagrams under illumination at a negative V_GR_ and a positive V_GR_, respectively. These schematics can be regarded as extensions of the band diagrams under dark conditions shown in Figure 2d. Under an illumination, carrier excitation occurs in both the CdSe QDs and WSe_2_. The photocurrent originating from WSe_2_ was observed at all V_GR_. In contrast, the photocurrent contribution from the CdSe QDs is strongly affected by the h‐BN tunneling barrier inserted at the CdSe QDs/WSe_2_ interface. At a negative V_GR_, the applied out‐of‐plane electric field narrows and lowers the tunneling barrier across h‐BN, facilitating exciton dissociation in the QDs. Photogenerated electrons tunnel through the h‐BN layer into WSe_2_, while the corresponding holes are extracted toward the graphene electrode. The resulting electron–hole separation exceeds the exciton binding energies in both the QDs and WSe_2_, leading to efficient spatial dissociation of excitons and enhanced interlayer carrier transfer. At a positive V_GR_, the direction of the electric field is opposite, which increases h‐BN tunneling barrier. As a result, the photogenerated electrons in CdSe QDs are no longer transferred to the WSe_2_ channel but are instead extracted toward the graphene, trapped, or recombined within the structure. The photogenerated holes are also expected to be trapped or recombined internally. As discussed later, we experimentally observed that no photocurrent is generated from the CdSe region at a positive V_GR_. Figure 3d,e shows the spectral responsivity measured at the drain and graphene electrode, respectively. The V_GR_ modulation is further evidenced by distinct responsivity peaks at near 760 and 550 nm, which correspond to excitonic transitions in WSe_2_ and CdSe QDs, respectively [44, 45]. The presence of the excitonic absorption peak of CdSe QDs implicates that photoexcited carriers in the QDs contribute to photocurrent through tunneling‐mediated charge transfer. In Figure 3d, the spectral responsivity measured at the drain reveals that the enhanced photoresponse at V_GR_ = −1 V originates from efficient dissociation of QD excitons and the subsequent carrier transfer into the WSe_2_ channel. When the graphene voltage is set to V_GR_ = 0 or 1 V, the spectral responsivity is dominated by the WSe_2_ excitonic peak, while the excitonic contribution from the CdSe QDs is significantly suppressed. Similarly, in spectral responsivity measured at the graphene electrode, the responsivity peak corresponding to the QD excitonic transition (∼550 nm) gradually decreases as V_GR_ increases from −1 to 1 V. This behavior reflects the suppression of tunneling‐mediated carrier extraction from the QDs as the effective tunneling barrier increases under positive graphene bias.

Scanning photocurrent mapping directly visualizes the spatial distribution of photocurrent generated via tunneling‐induced charge transfer, demonstrating its strong dependence on the applied graphene voltage. Figure 3f–h shows the corresponding photocurrent mapping at V_GR_ = −1, 0, and 1 V, respectively. A 532 nm laser was used for photocurrent mapping because its photon energy lies close to the excitonic absorption peak of CdSe QDs, facilitating a strong photocurrent signal that reflects their spatial absorption distribution. The photocurrent at the drain was measured under various graphene voltages using a 532 nm laser at an irradiance of 0.37 W/cm^2^ with a fixed V_DS_ = 0 V. At V_GR_ = −1 V, the photocurrent signal is predominantly localized in the WSe_2_/h‐BN/CdSe QDs/graphene junction (overlap region). This spatial confinement arises because QD‐derived electrons are rapidly extracted into graphene before undergoing lateral diffusion or recombination. In contrast, no significant photocurrent is observed in the WSe_2_/metal contact regions due to the suppressed carrier transfer across the h‐BN barrier. Quantitatively, the photocurrent in the overlap region is ∼1.9 × 10^−10^ A, and the photocurrent in the WSe_2_/metal contact regions reaches ∼2 × 10^−11^ A at the V_GR_ = −1. The photocurrent shifts predominantly to the WSe_2_/metal contact regions with magnitudes of ∼ 5 × 10^−11^ A at V_GR_ = 0 and 1 V, while the photocurrent signal from the overlap region decreases by an order of magnitude relative to that at −1 V. Figure 3i–k indicate the corresponding photocurrent mapping generated from the graphene current to V_GR_ = −1, 0, and 1 V. Unlike the photocurrent mapping based on the drain current (I_DS_), the photocurrent signals at the WSe_2_/metal contact regions are significantly suppressed. This behavior arises because the electric field established by the applied V_GR_ promotes exciton dissociation in the QDs. The overlap region of the WSe_2_/h‐BN/CdSe QDs/graphene junction in Figure 3i exhibits strong photogenerated carrier separation at V_GR_ = −1 V. The device exhibited no detectable photocurrent signal at V_GR_ = 0 and 1 V, indicating that photogenerated carriers in the QDs recombine instead of being separated, as shown in Figure 3j,k. This redistribution provides clear evidence that the V_GR_ effectively modulates the QD‐graphene tunneling barrier, controlling the direction and efficiency of photocarrier extraction. Such voltage‐dependent tunability highlights the dynamic control of charge transfer in mixed‐dimensional heterostructures.

### 2.4 Photodetector Performance and Application

**FIGURE 4:**
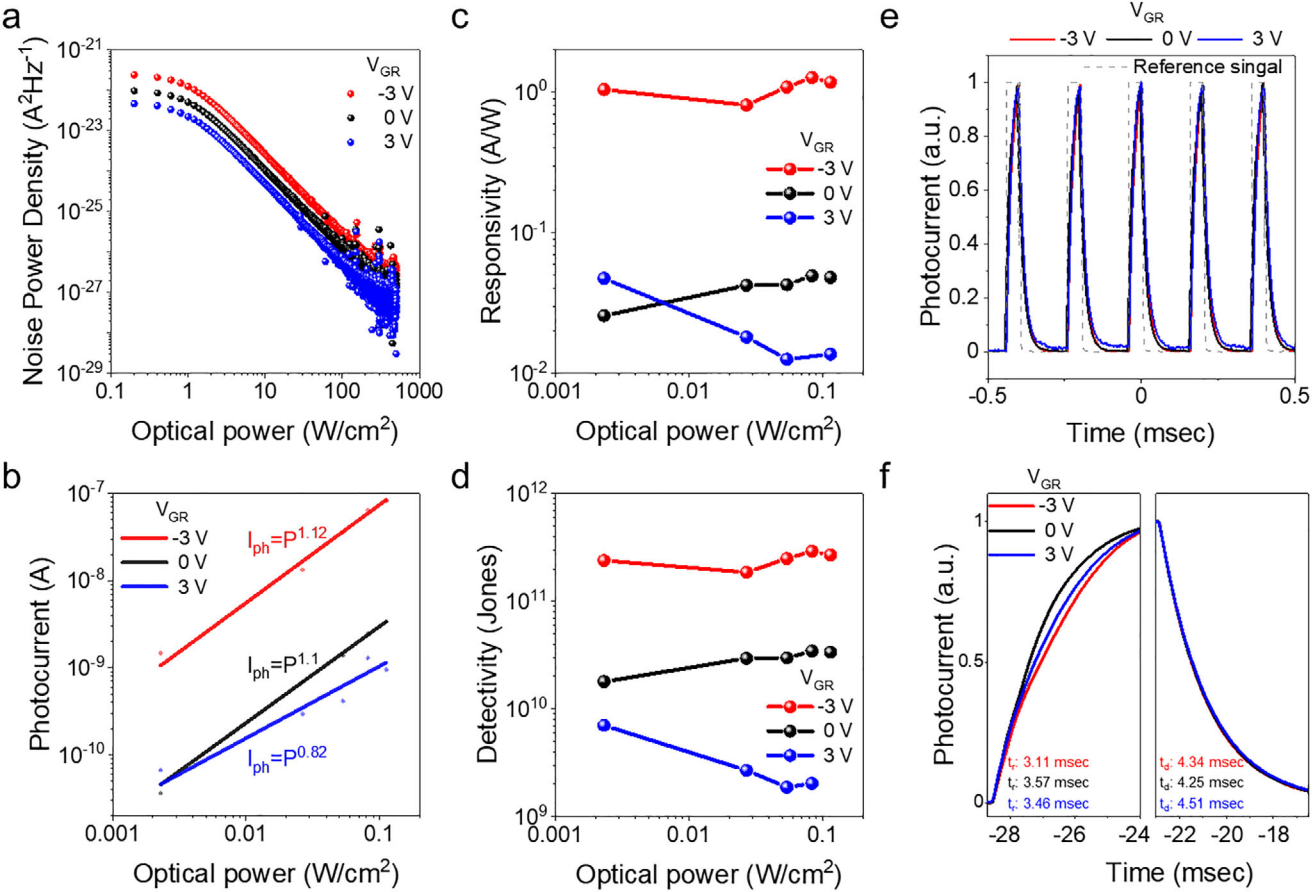
(a) Noise power spectral density (A^2^/Hz) versus frequency (Hz) measured at room temperature for V_GR_ = −3, 0, and 3 V. (b) Photocurrent–power relation under 405 nm illumination (0.002–0.11 W/cm^2^) exhibiting power exponents γ = 1.12, 1.10, and 0.82 at V_GR_ = −3, 0, and 3 V, respectively. (c,d) Responsivity and Detectivity plots at V_GR_ = −3, 0, and 3 V. (e,f) Time‐resolved photocurrent response of the device, (f) rising and decay time.

Carrier number fluctuations, arising from trapping and de‐trapping processes at the CdSe QD/h‐BN/WSe_2_ interface as well as from carrier scattering, represent the primary source of noise [46, 47]. In tunneling‐based devices, defect states residing within the bandgap act as trap centers that intermittently capture and release tunneling carriers [48, 49]. These trapping and de‐trapping phenomena dynamically modulate the effective tunneling barrier, inducing fluctuations in the carrier population of the graphene. Moreover, charged carriers residing within the insulator are known to influence the tunneling barrier significantly [49]. In nanoscale devices, the stochastic occupation of only a limited number of active traps leads to perceptible variations in the tunneling current. Accordingly, carrier tunneling, which is mediated by trap‐assisted capture and de‐trap processes at the interface, has been identified as the dominant cause of the observed 1/f noise [47]. As shown in Figure 4a, the noise power spectral density at 1 Hz decreases from 1.2 × 10^−22^ A^2^/Hz at V_GR_ = −3 V to 2.2 × 10^−23^ A^2^/Hz at 3 V, indicating suppressed trap‐induced fluctuations as the tunneling barrier increases under positive voltages. Conversely, when the tunneling barrier is lowered under negative graphene bias, enhanced trap‐assisted tunneling increases carrier number fluctuations, resulting in an increased noise level. Figure 4b shows the device photocurrent as a function of optical power at various graphene voltages. The photocurrent follows a power‐law relation with incident power (I_ph_ ∝ P_eff_^γ^) [50, 51]. We observed that the exponent γ strongly depends on the graphene voltage in the heterostructure devices. Table 2 summarizes the performance comparison of WSe_2_‐based photodetectors and related hybrid systems. Unlike conventional photogating‐based devices that rely on trap‐assisted gain, our device enables bias‐controlled modulation of carrier injection through a tunneling barrier, providing a distinct approach for engineering photoresponse characteristics. At V_GR_ = −3 and 0 V, we obtain super‐linear responses of γ = 1.12 and 1.10, respectively. Such γ > 1 behavior is attributed to carrier‐assisted injection across a voltage‐tunable barrier [52]. The superlinear photoresponse (γ > 1) can be attributed to the combined effects of enhanced carrier injection and suppression of recombination processes. At high illumination power, trap states become saturated, effectively reducing recombination channels and enabling more efficient carrier collection. This effect is further amplified under negative graphene bias, where the tunneling barrier is lowered, resulting in increased carrier injection from the quantum dots. Under high optical power, the graphene/CdSe QDs hybrid receives photon energy, which broadens the high‐energy portion of the electron distribution and enhances the probability of carriers overcoming the interfacial barrier in a non‐linear manner (photo‐thermionic/thermionic injection) [53, 54]. This mechanism is well established in graphene‐based Schottky and van der Waals junctions. The interaction between the effective electronic temperature caused by photon energy and barrier selectivity results in the injected current showing super‐linear power expansion. In our mixed‐dimensional device, a similar mechanism governs carrier transfer from CdSe QDs into the WSe_2_ channel when the graphene voltage effectively lowers or thins the tunneling barrier. The resulting non‐linear increase of injection probability with optical power accounts for the observed γ > 1 at V_GR_ ≤ 0 V. In contrast, the exponent decreases to γ = 0.82 at V_GR_ = 3 V and indicates sub‐linear behavior, suggesting that the photoresponse is dominated by WSe_2_ carrier due to suppression of charge carrier injection with a higher effective barrier. In this regime, γ < 1 typically occurs when trap‐ or recombination‐mediated pathways dominate, the progressive occupation of light‐responsive electronic states limits the generation of additional carriers [55]. Figure 4c,d presents detectivity (D^*^) and responsivity as a function of optical power from 0.002 to 0.11 W/cm^2^ using a 405 nm laser at different graphene voltages. Responsivity is defined as photocurrent as a function of incident light power and is expressed by the equation R = I_ph_/P_eff_, where I_ph_ is the photocurrent and P_eff_ (W/cm^2^) = optical power (W)/laser beam area (cm^2^). The following equation expresses detectivity (D^*^) $D^{*}=\frac{R\sqrt{AB}}{i_{N}}\;$: A = active area, B = bandwidth, and *i_N_* = noise. We calculated the detectivity by considering both shot noise and flicker noise. Shot noise was obtained from the dark current, extracted as $i_{N}=\sqrt{2eI_{dark}}\;$. To obtain the noise spectrum density for the flicker noise, a fast Fourier transform (FFT) was performed on the dark current trace. The 1 Hz noise component was then used as the flicker noise current. Figure 4c indicates that responsivity is highest at V_GR_ = −3 V (1.1 A/W), while values at 0 V (0.04 A/W) and 3 V (0.023 A/W) are substantially lower. D^*^ decreases sharply for higher V_GR_, following values of 2.4 × 10^11^, 2.9 × 10^10^, and 3.4 × 10^9^ Jones at −3, 0, and 3 V, respectively, as shown in Figure 4d. To quantitatively evaluate the photodetector performance, the external quantum efficiency (EQE), short‐circuit current (Isc), and open‐circuit voltage (Voc) were extracted under different VGR conditions and are summarized in Figure S11. The EQE was calculated from the measured responsivity (R) using the relation $\mathrm{EQE}\;=\frac{Rhc}{q\lambda }\;$ [26]. The device exhibits strong V_GR_ dependence, where both Isc and V_OC_ are significantly enhanced under negative graphene bias. At V_GR_ = −3 V, the device shows a V_OC_ of 1.6 V and an Isc of ∼4.5 nA. This pronounced enhancement at negative graphene bias indicates that photogenerated carriers in the CdSe QDs actively contribute to the photocurrent via tunneling‐mediated charge transfer. Figure 4e shows the transient photoresponse of the WSe_2_/h‐BN/CdSe QDs/graphene heterostructure under periodic illumination, for 15 Hz pulsed light with a wavelength of 405 nm and a power of 0.11 W/cm^2^, revealing both the rising and decay behaviors at different graphene voltages. The detailed analysis in Figure 4f quantifies the corresponding time constants. The rising time (τ_r_) decreases under a negative graphene voltage (3.11 msec at V_GR_ = −3 V) compared with a positive voltage (3.46 msec at V_GR_ = 3 V), indicating that the lowered Fermi level at negative voltage reduces the tunneling barrier and facilitates faster carrier extraction from the QDs. In contrast, the decay time (τ_d_) remains nearly unchanged (4.34–4.51 msec) regardless of graphene voltage, suggesting that the recombination or trap‐release process is dominated by intrinsic material dynamics rather than external voltage modulation. We also investigated the transient photoresponse under 532 nm illumination, as shown in Figure S10. Under 532 nm excitation, both rise and decay times were significantly longer than those measured under 405 nm illumination. These results indicate that the transient response strongly depends on the excitation energy, owing to the wavelength‐dependent optical absorption profile and exciton dissociation dynamics. In the case of 405 nm excitation, the higher photon energy promotes carrier excitation to higher energy states and facilitates carrier extraction, resulting in faster charge separation and a shorter response time.

**TABLE 2:** Comparison of performance metrics of WSe_2_‐based and mixed‐dimensional photodetectors.

Device structure	Type	Bias condition	Responsivity (A/W)	Mechanism	Refs.
WSe_2_ monolayer	2D	V_DS_ = 2 V, V_GS_ = 0 V	0.175 A/W (550 nm)	Photogating	[56]
WSe_2_/ReS_2_	2D‐2D	self‐powered	0.08–0.29 A/W (785 nm)	Charge transfer	[57]
Graphene/WSe_2_/Graphene	2D‐2D	V_B_ = 0.6 V	0.12 mA/W (1500 nm)	Photo‐thermionic	[52]
hBN/Graphene/MoS_2_/Graphene	2D‐2D		1.1 A/W (633 nm)	Charge transfer	[58]
WSe_2_/graphene/MoTe_2_	2D‐2D	V_DS_ = 0 V	40.84 mA/W (550 nm)	Charge transfer	[59]
MoS_2_/graphene/WSe_2_	2D‐2D	V_GS_ = −60 V V_DS_ = 1 V	2.6 A/W (532 nm)	Photovoltaic	[60]
h‐BN/WSe_2_/DDAB‐CsPbX_3_	2D‐0D	V_DS_ = 0.3 V V_GS_ = −1.5 V	90 A/W – WSe_2_ 2000 A/W – WSe_2_/DDAB CsPbX_3_ (405 nm)	Photogating	[61]
MoS_2_/CdSe	2D‐0D	V_GS_ = −40 V V_DS_ = 0 V	100 A/W – MoS_2_ 400 A/W – MoS_2_/CdSe (580 nm)	Charge transfer	[62]
ReS_2_/CdSe	2D‐0D	V_DS_ = 3 V	26 A/W – ReS_2_ 654 A/W – ReS_2_/CdSe (589 nm)	Charge transfer	[63]
This work (WSe2/h‐BN/CdSe QD/Graphene)	2D‐0D	V_GR_ = −3 V	1.1 A/W (405 nm)	Charge transfer	
		V_GR_ = 0 V	0.04 A/W (405 nm)		This work
		V_GR_ = 3 V	0.023 A/W (405 nm)		

## 3 Conclusion

In summary, we demonstrated that the tunneling barrier across a 2.5 nm thick h‐BN layer in WSe_2_/h‐BN/CdSe QDs/graphene heterostructures can be dynamically tuned by graphene voltage, enabling controlled charge transfer between zero‐ and two‐dimensional materials. Time‐resolved photoluminescence measurements revealed a pronounced reduction in carrier lifetime for thin h‐BN barriers, confirming efficient tunneling‐mediated carrier extraction compared with thick‐barrier structures. Electrical transport analyses showed clear V_GR_‐dependent modulation of band alignment, with Fowler–Nordheim transition voltages decreasing from 23.3 to 16.7 V as the graphene voltage was varied from +3 to −3 V, indicating a substantial lowering of the effective tunneling barrier. Independent photocurrent mapping at the drain and graphene electrodes directly visualized the redistribution of photocarriers, revealing a bias‐dependent transition between graphene‐localized carrier extraction and source‐drain‐dominated transport. Under negative V_GR_, the device exhibited enhanced photoresponse characterized by a super‐linear power‐law exponent (γ ≈ 1.12), while a sub‐linear response (γ ≈ 0.82) was observed at positive bias, consistent with barrier‐controlled injection mechanisms. Furthermore, the responsivity and detectivity reached maximum values of approximately 1.1 A/W and 2.4 × 10^11^ Jones, respectively, under negative graphene voltage, representing an order‐of‐magnitude enhancement compared with positive‐bias operation. Noise spectral analysis showed a corresponding reduction in low‐frequency noise with increasing tunneling barrier height, corroborating the role of trap‐mediated tunneling processes. Collectively, these quantitative results establish a versatile and electrically programmable tunneling platform that overcomes the intrinsic band‐alignment limitations of conventional van der Waals heterostructures. This work advances the fundamental understanding of graphene‐biased tunneling in mixed‐dimensional 0D–2D hybrids and provides practical design guidelines for next‐generation optoelectronic and quantum devices with tunable charge–transfer pathways and performance metrics. Unlike conventional photodetectors with fixed operating mechanisms, our device enables active control of the tunneling barrier via graphene bias, allowing dynamic modulation of carrier injection and photoresponse. This functionality provides an additional degree of freedom for designing tunable optoelectronic systems beyond simple detection.

## 4 Experimental Section

### 4.1 Material for Synthesis

Cadmium oxide (99.99%), selenium powder (99.99%), oleic acid, oleylamine (70%), sulfur powder (99.998%), zinc acetate (99.999%), trioctylphosphine oxide (TOPO 90%), and 1‐octadecene (ODE 90%) were all purchased rom Sigma–Aldrich. Trioctylphosphine (TOP 97%) was purchased from Stream Chemicals. All chemicals were used without further purification.

### 4.2 Synthesis of CdSe/ZnS

0.5 m cadmium oleate was prepared by mixing 10 mmol cadmium oxide (CdO), 20 mmol oleic acid, and 14 mL ODE, and the mixture was degassed and heated to 240°C for 30 min. Subsequently, 5.7 mmol TOPO was mixed with 50 mmol ODE and 3 mL of the prepared Cd (Oleate)_2_ solution. The mixture was then degassed at 100 C for 60 min and subsequently heated to 300 C. Next, 6 mL of oleylamine and 8 mL of 1 m TOP‐Se were sequentially injected into the solution. The reaction was maintained at 300 C for 10 min to promote the growth of a desirable CdSe core. To grow ZnS shell, 2 mL of 0.1 m zinc oleate and 0.1 m ODE‐S were sequentially hot‐injected into the desirable core solution. The quantum dots were separated from the crude using size‐selective precipitation with acetone, and the final QDs were dispersed in hexane.

### 4.3 Spectroscopy Measurement

The absorption spectra were measured in an absorption spectrophotometer (Cary 5000 UV–vis–NIR, Agilent Technologies) using a 1 cm cuvette. Steady‐state photoluminescence spectra of the QDs were recorded using a Horiba‐Fluoromax 4 spectrophotometer at 400 nm excitation. X‐ray Photoelectron spectroscopy (XPS) measurements were performed using an ESCALAB 250 Xi spectrometer (Thermo Scientific) with an Al K_α_ monochromatic X‐ray source (1486.6 eV). The binding energy was calibrated based on the carbon C 1s peak at 284.6 eV.

### 4.4 Device Fabrication Processes

Bulk WSe_2_ (HQ‐graphene) was exfoliated on SiO_2_/Si substrate, and WSe_2_ was stacked by dry transfer on a pre‐patterned SiO_2_/Si substrate using a photolithography process. E‐beam lithography was used to fabricate the electrode patterns, and an e‐beam resistor (PMMA, 950K) was used. The metal was deposited as Ti (10 nm)/Au (50 nm) by an e‐beam evaporator (SNR‐200). Metal lift‐off was performed in acetone at 60°C. Then, the devices were annealed at 120°C for 30 min under an N_2_ atmosphere in a glove box to minimize contact resistance at the metal/semiconductor interface.

### 4.5 Electrical and Optoelectrical Measurements

The transfer and output curves shown in Figure S8 were measured in dark and under light illumination using a Keithley 4200A source meter and a 405 nm laser as the light source, which was incident onto the devices in a glovebox. An in‐house LabVIEW program controlled the Keithley 2636B, and the scanner was connected via an RS232 cable.

### 4.6 Power Spectral Density Measurements

After synchronizing the Keithley 2636B with the LabVIEW program, 1000 Hz sampling and 5 s current tracing were performed. The noise power densities (A^2^/Hz) were extracted from the raw noise current trace data using a fast Fourier transform. The spectral noise current (A/Hz^1/2^) was obtained by taking the root of the noise power density. To calculate the detectivity using the noise current, the response for a 1 Hz modulation was extracted.

### 4.7 Time‐Resolved Photocurrent Measurements

For time‐resolved photocurrent measurements, a 15 Hz pulsed signal was applied to a 405 nm laser source using a function generator (GW Instek, SFG‐2110). The drain voltage was supplied through a preamplifier (Stanford Research Systems, SR570), while the gate voltage was controlled via a Keithley 4200 source meter. The output terminal of the SR570 was connected to an oscilloscope (Agilent Technologies, MSO‐X 2024A) to record the on‐off temporal photocurrent response.

### 4.8 TRPL Measurements

The TRPL study was carried out using a confocal microscope (MicroTime‐200, Picoquant, Germany). The lifetime measurements were performed at the Korea Basic Science Institute (KBSI), Daegu Center, Korea. A single‐mode pulsed diode laser (470 nm with a pulse width of ∼30 ps and an average power of ∼150 nW operating at 2 MHz repetition rate) was used as an excitation source. A dichroic mirror (490 DCXR, AHF), a long‐pass filter (HQ500lp, AHF), a 100 µm pinhole, a bandpass filter (500–650 nm, Thorlabs), and a single photon avalanche diode (PDM series, MPD) were used to collect emission from the samples. A 60× (water) objective (NA 1.2) was used for laser illumination to the perovskite films on a glass. A time‐correlated single‐photon counting system (PicoHarp‐300, PicoQuant GmbH, Germany) was used to count emission photons. PL lifetime images consisting of 200 × 200 pixels were recorded using the time‐tagged time‐resolved (TTTR) data acquisition method. Exponential function fittings for the obtained PL decays were performed using Symphotime‐64 software (Ver. 2.2).

## Conflicts of Interest

The authors declare no conflicts of interest.

## Supporting information




**Supporting File**: smll73645‐sup‐0001‐SuppMat.docx.

## Data Availability

The data that support the findings of this study are available on request from the corresponding author. The data are not publicly available due to privacy or ethical restrictions.
