# Supplementary material for: Electrically Tunable Tunneling and Spectral Response in WSe2/h‐BN/CdSe/Graphene Heterostructure
Source: Small. 2026 May 6;22(35):e73645. doi: 10.1002/smll.73645 (PMC13288855; doi:10.1002/smll.73645)
Supplement: Supplementary file 1 — Supporting File: smll73645‐sup‐0001‐SuppMat.docx. [file SMLL-22-e73645-s001.docx]

Supporting Information

**Electrically tunable tunneling and spectral response in WSe₂/h-BN/CdSe/Graphene heterostructure**

Sang-Hyeon Lee^1^, Justice Agbeshie Teku^1^, Min-Hye Jeong^1^, Jae-Hyeon Ahn^1^, Weon-Sik Chae^2^, Dohyun Kwak^3^*, and Jong-Soo Lee^1^*

^1^Department of Energy Science & Engineering, Daegu Gyeongbuk Institute of Science and Technology (DGIST), Daegu 42988, Republic of Korea

E-mail: jslee@dgist.ac.kr

^2^Division of Materials Science, Korea Basic Science Institute, Daejeon 34133, Republic of Korea

^3^Division of Nanotechnology, Daegu Gyeongbuk Institute of Science and Technology (DGIST), Daegu 42988, Republic of Korea

E-mail: ckd3138@dgist.ac.kr


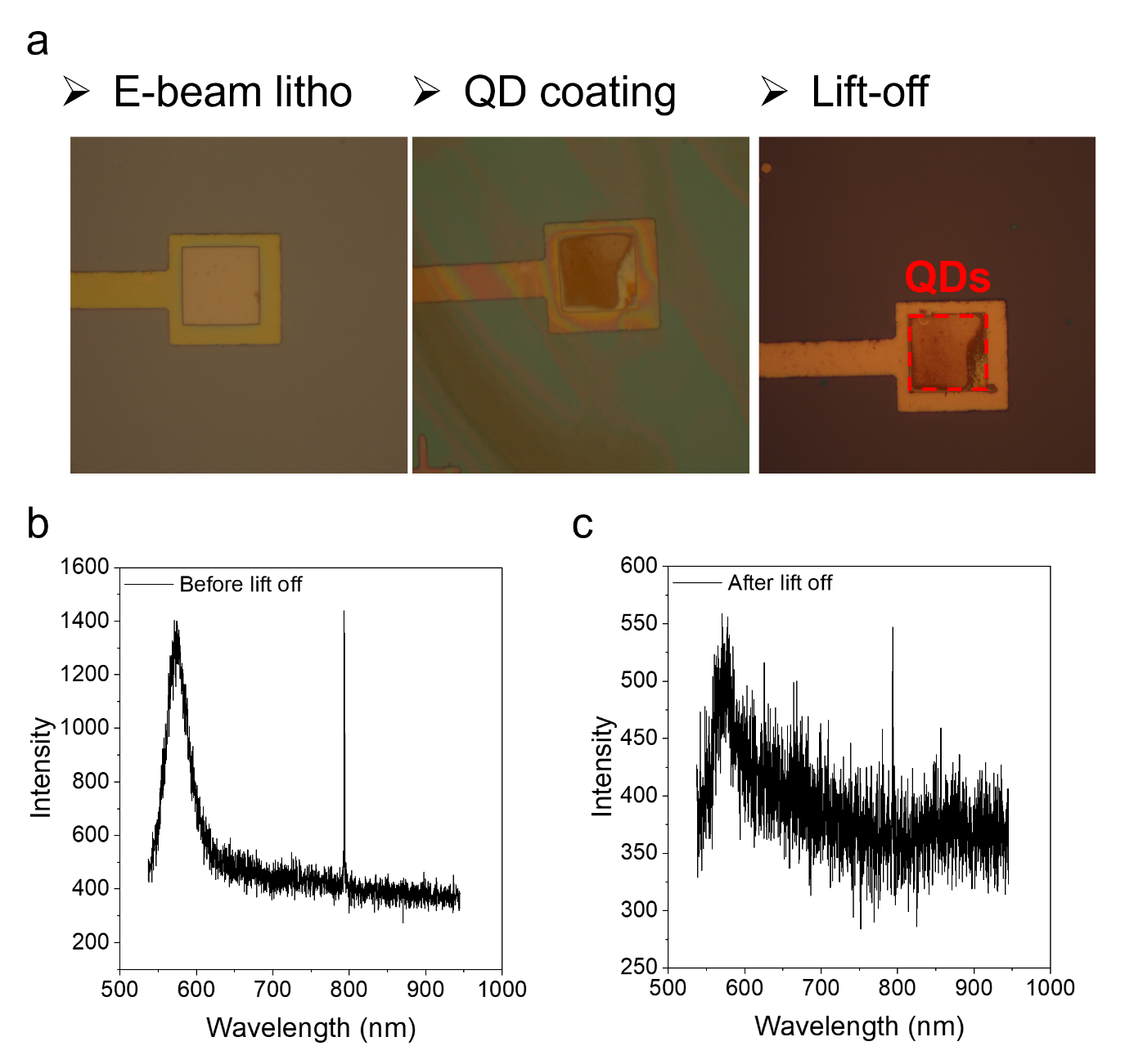
**Figure S1** (a) Optical microscope images showing each fabrication step of the QD patterning process: e-beam lithography, QD coating, and lift-off. (b,c) Photoluminescence (PL) spectra of CdSe/ZnS QDs film measured before and after the lift-off process under 532 nm excitation, confirming the successful definition of the patterned QD region.


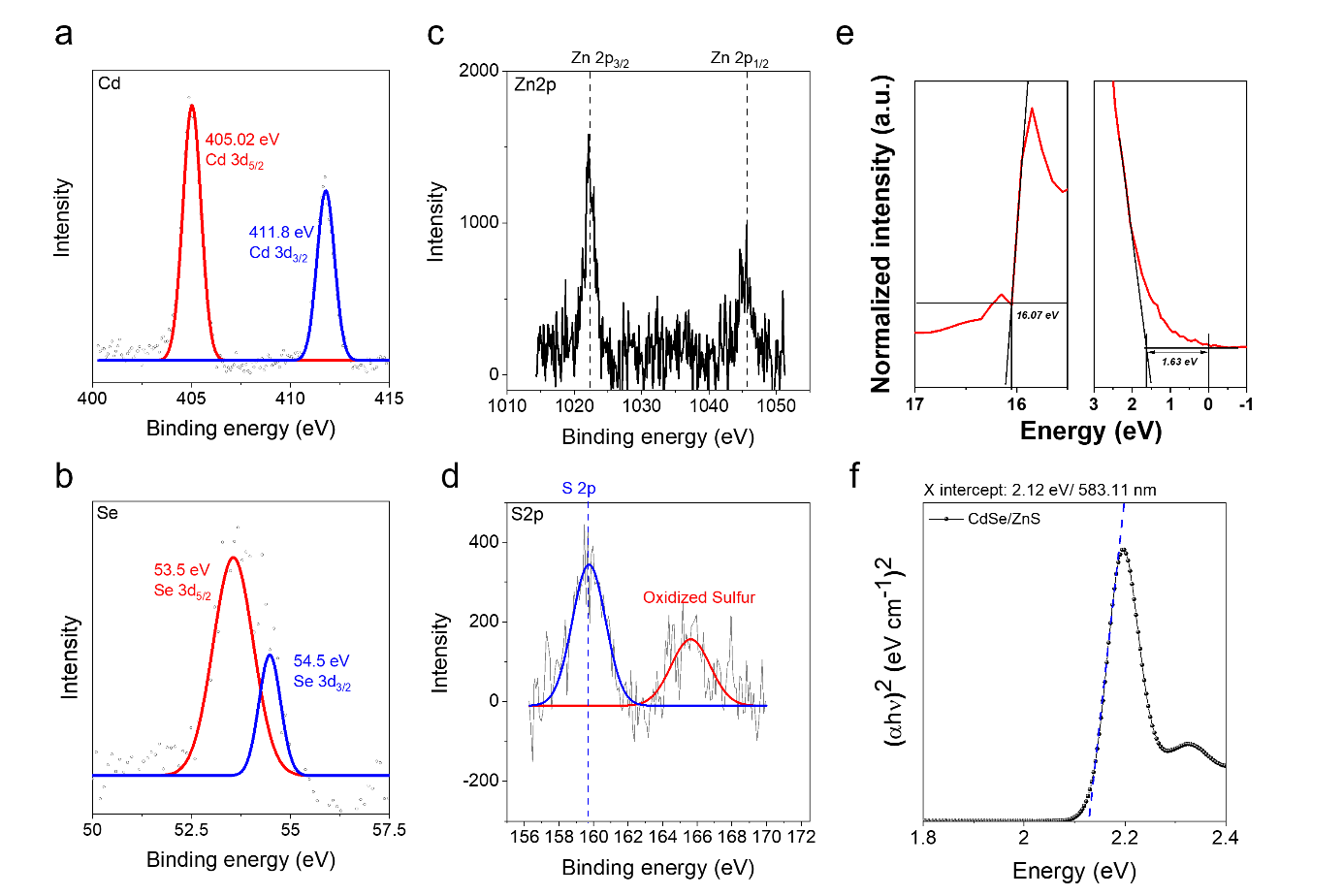


**Figure S2** (a,b) XPS spectra of Cd 3d and Se 3d core levels showing Cd²⁺ (411.8 eV) and Se²⁻ (54.5 eV) states in CdSe. (c,d) XPS spectra of Zn 2p and S 2p confirming ZnS shell formation and partial surface oxidation of sulfur. (e) UPS spectra of CdSe/ZnS QDs showing valence band maxima of 1.63 eV and 1.60 eV. (f) Tauc plot derived from the absorption spectrum, giving an optical bandgap of 2.12 eV (≈ 583 nm).


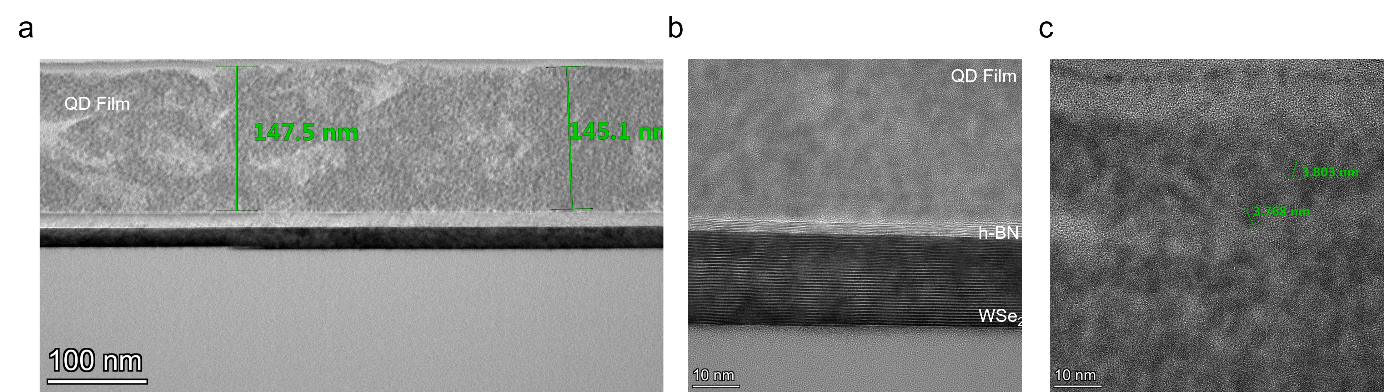


**Figure S3** Cross-sectional TEM images of the WSe_2_/h-BN/CdSe QD heterostructure. (a) Low-magnification TEM image showing a uniform CdSe QD film (~145–150 nm) deposited on the layered heterostructure. (b) High-resolution TEM image of the WSe_2_/h-BN interface, revealing atomically stacked layers with a clean and well-defined boundary. (c) Magnified TEM image of the QD film, confirming closely packed nanocrystals with an average size of ~3.8 nm.


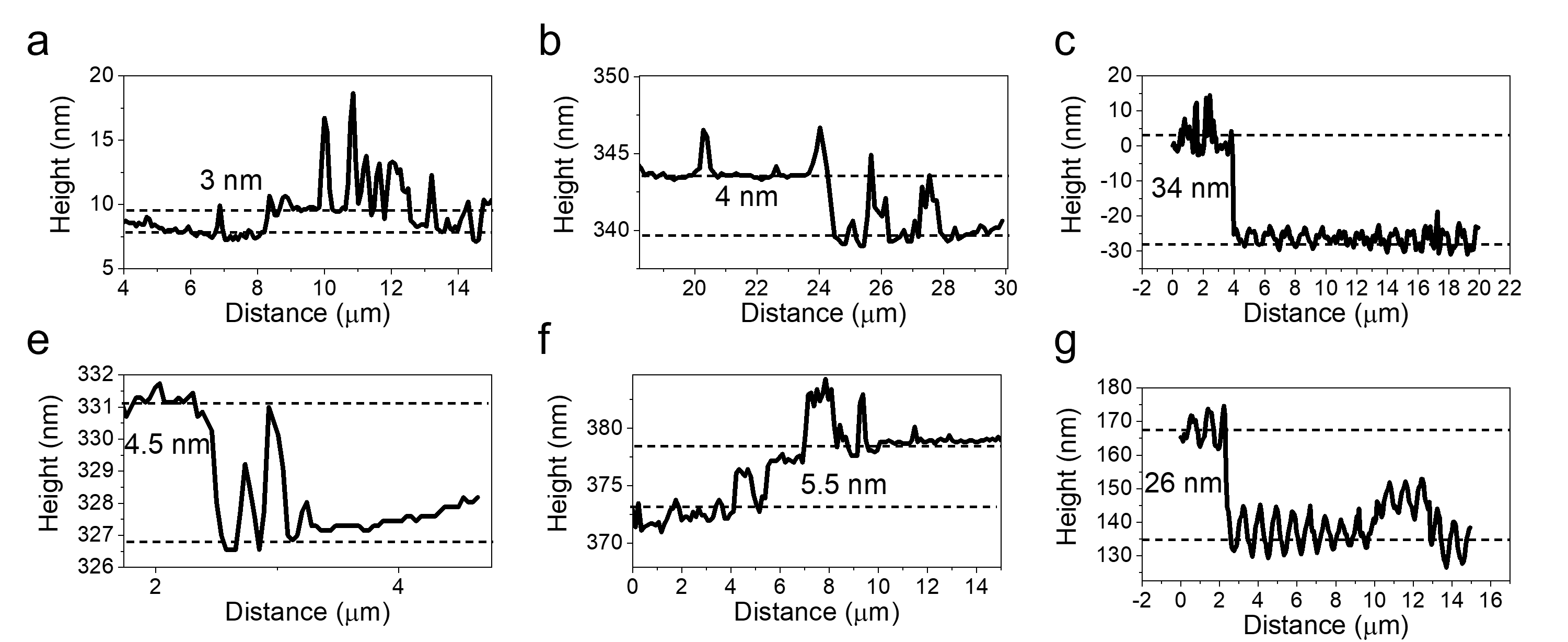


Figure S4 AFM data of thin h-BN thickness


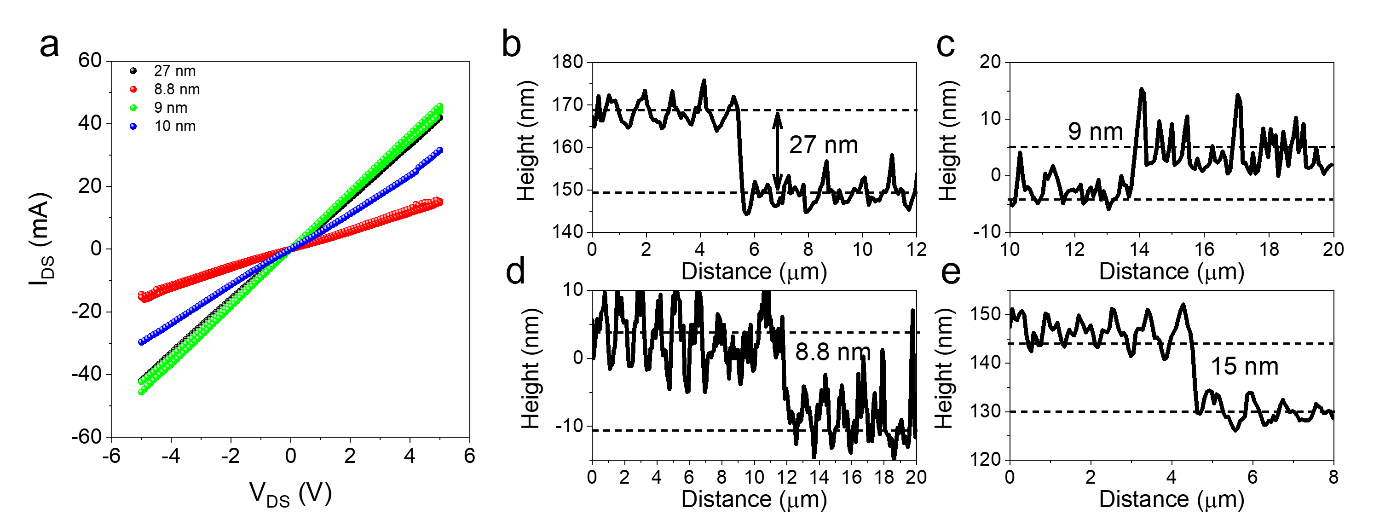


Figure S5 (a) Output characteristics (I_DS_-V_DS_) of graphene channels with varying thicknesses. (b–e) AFM height profiles of representative graphene flakes showing measured thicknesses of 27, 9, 8.8, and 15 nm, respectively.


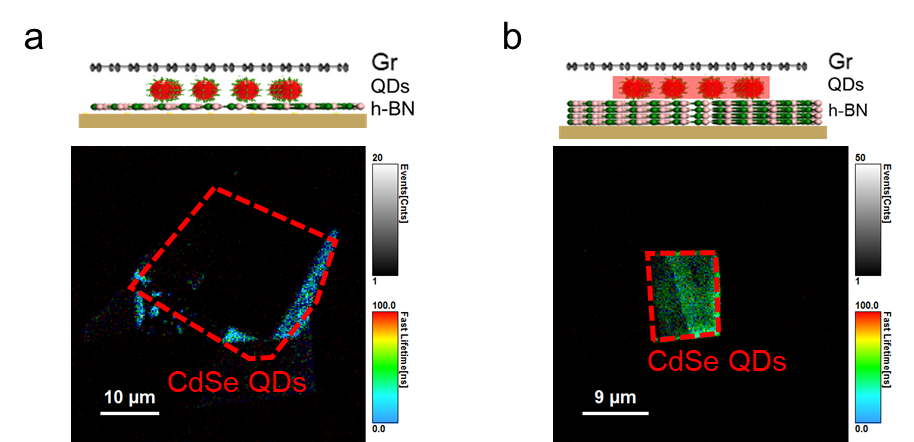


**Figure S6** (a,b) TR-PL mapping images of CdSe QDs with thin (~3 nm) and thick (~30 nm) h-BN tunnel barriers in /h-BN/QD/graphene heterostructures, excited by a 470 nm laser.


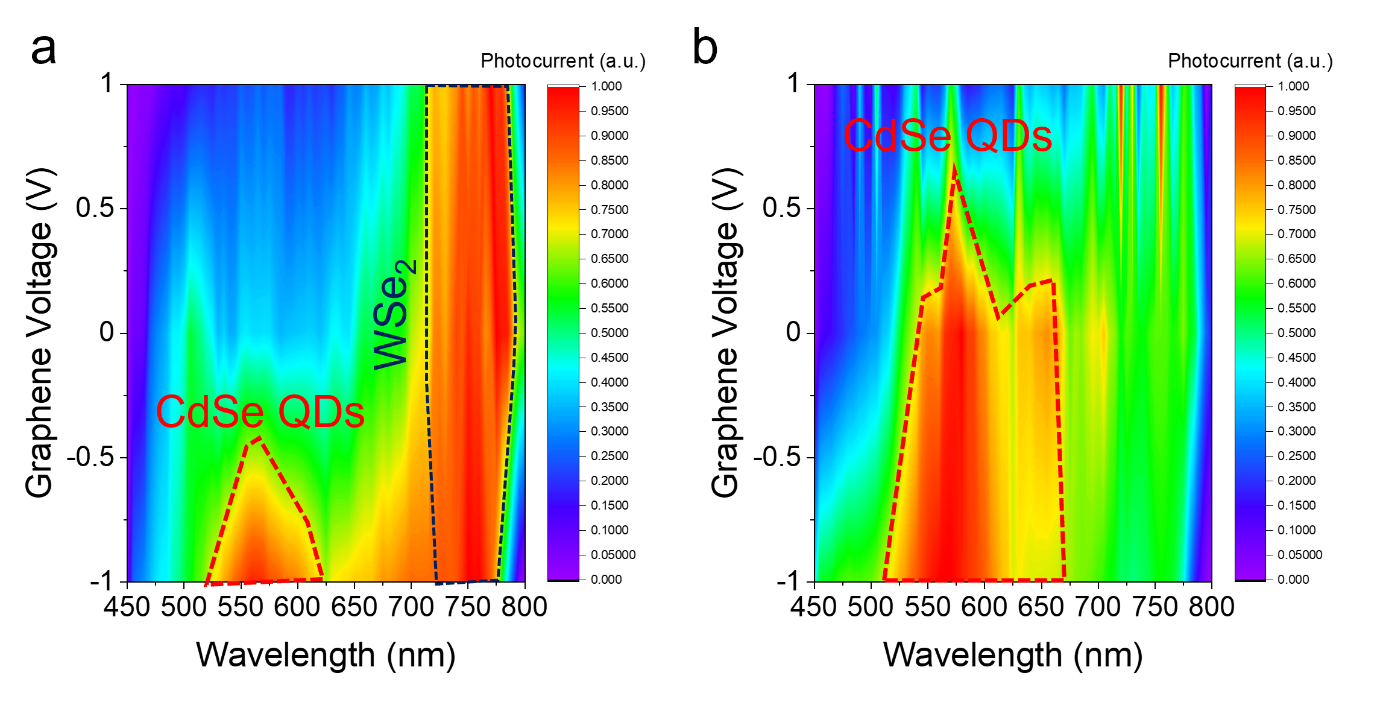


Figure S7 (a,b) Spectral responsivity color maps of photocurrent as a function of graphene voltage (−1 to +1 V) and illumination wavelength (450–800 nm), showing drain (a) and graphene (b) photocurrent.


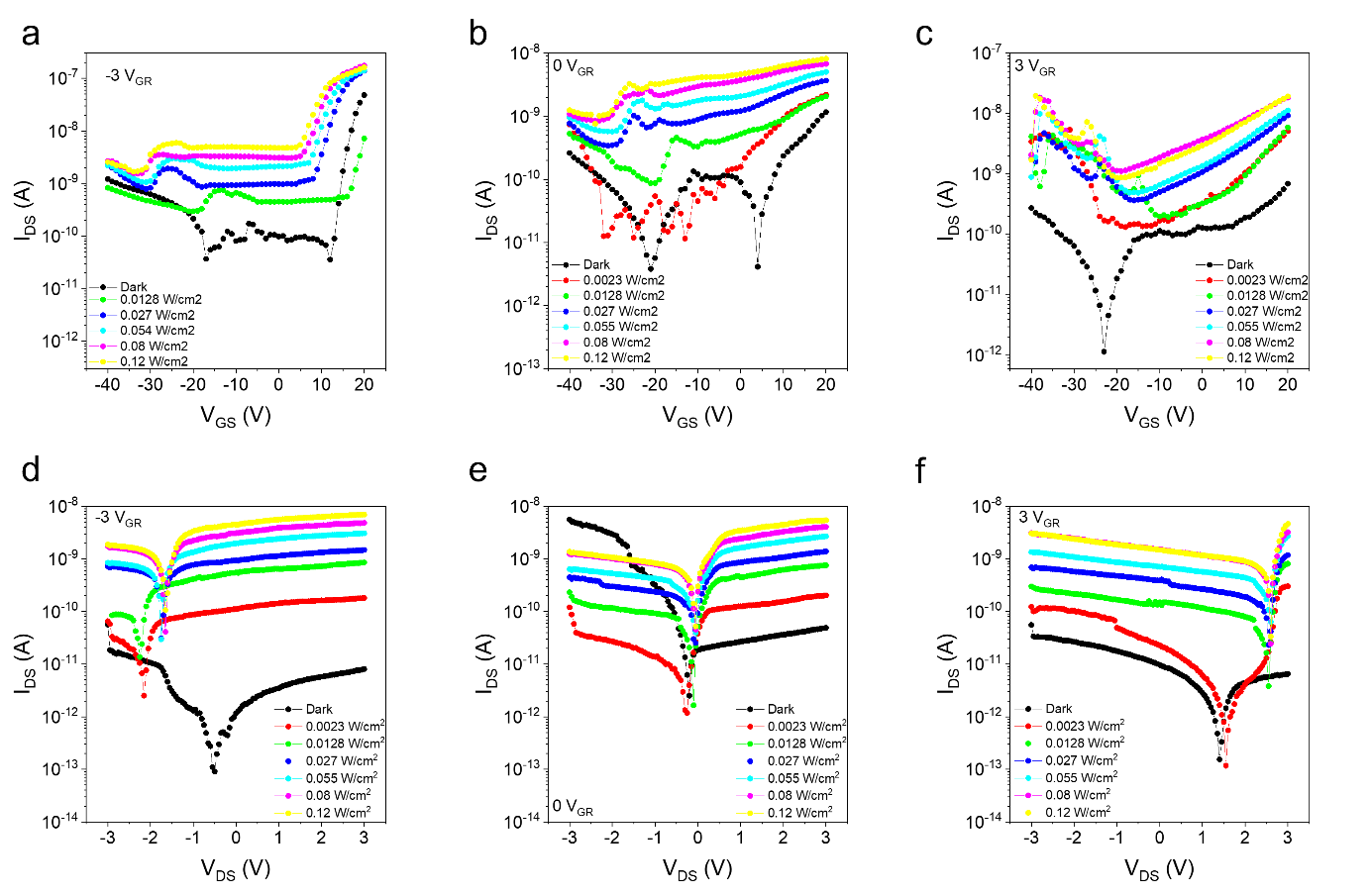
**Figure S8** (a–c) Transfer curves (I_DS_–V_GS_) measured under various optical powers (0.002–0.12 W cm⁻²) for V_GR_ = −3 (a), 0 (b), and 3 V (c). (d–f) Corresponding output curves (I_DS_–V_DS_) under various optical powers (0.002–0.12 W cm⁻²). Measurements were conducted using a 405 nm laser at room temperature.

**
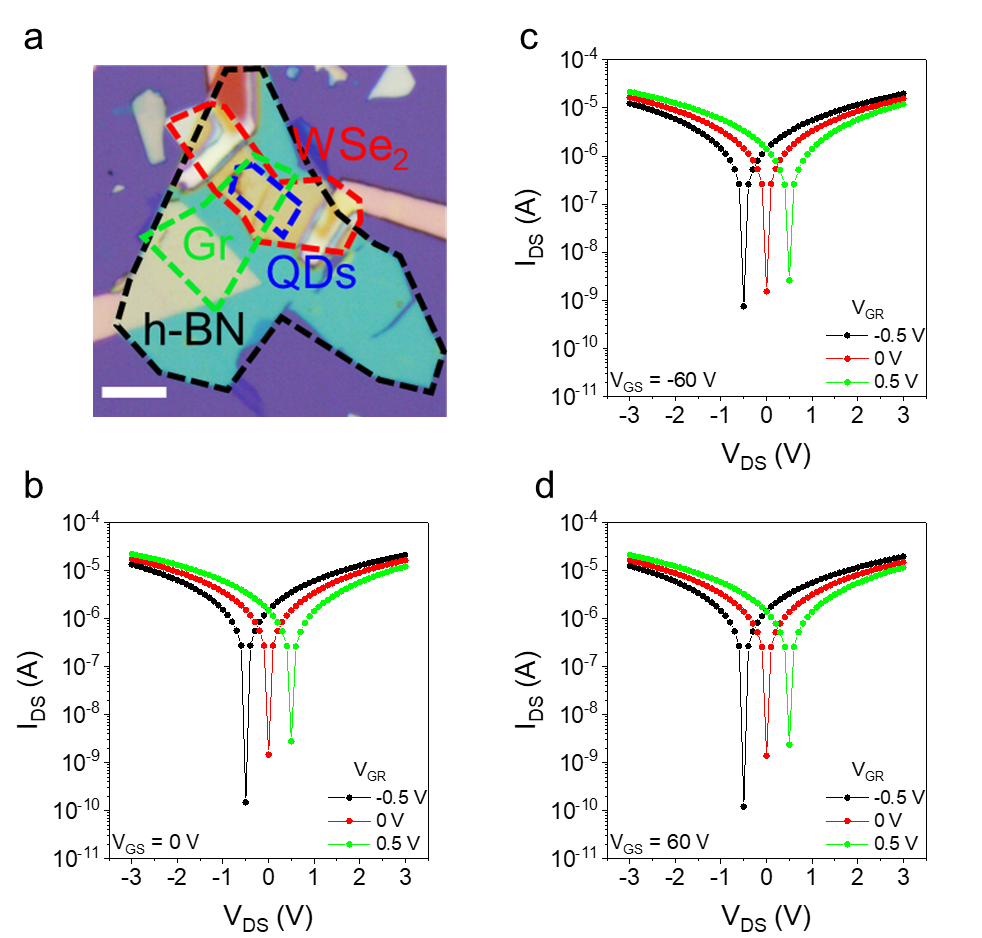
**

**Figure S9** (a) Optical microscopy (OM) image of the WSe_2_/h-BN/CdSe QDs heterostructure, scale bar 10 mm. (b–d) Output characteristics (I–V curves) of the device measured at V_GS_ = −60 V, 0 V, and 60 V, respectively, under different graphene bias conditions. The current–voltage curves show a clear shift depending on the graphene bias, indicating strong bias-dependent modulation.


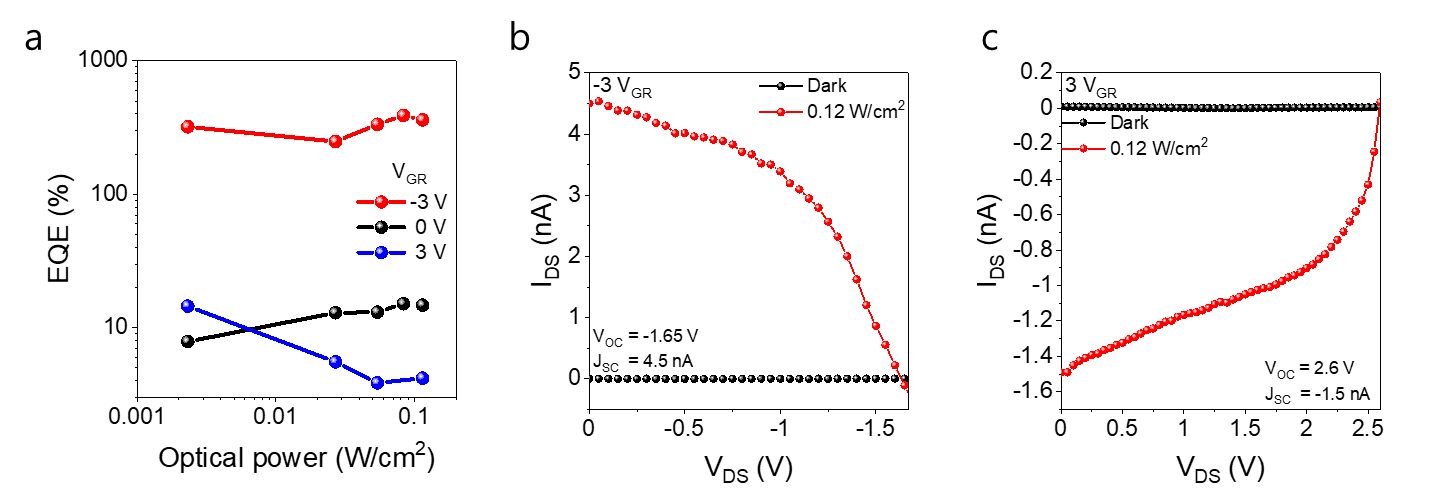


Figure S10 (a) Responsivity and detectivity of the device measured under 532 nm illumination at different graphene bias conditions (V_GR_ = −3, 0, and 3 V). (b) Time-resolved photocurrent response of the device under periodic illumination at 532 nm. (c) Rising and decay times corresponding to the transient photoresponse.


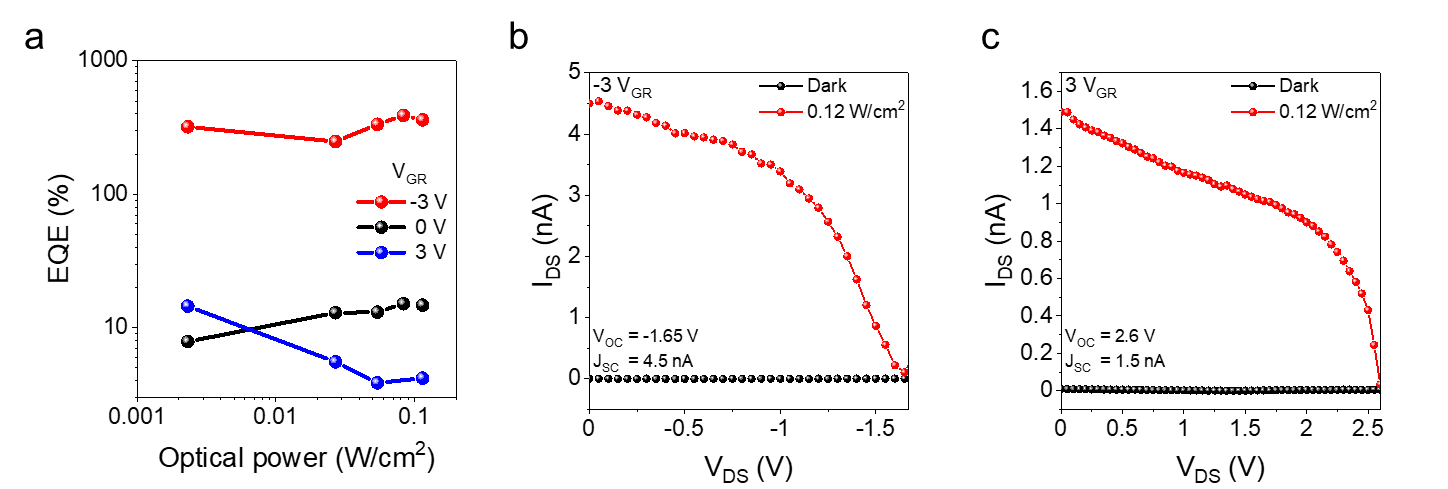


**Figure S11** (a) External quantum efficiency (EQE) of the device measured under 405 nm illumination at different graphene bias conditions (V_GR_ = −3, 0, and 3 V). (b,c) Short-circuit current (I_SC_) and open-circuit voltage (V_OC_) characteristics of the device measured at V_GR_ = −3 V and 3 V, respectively.
